# The Effectiveness of Probiotics on Oral Health During Adult Orthodontic Treatment With Fixed Appliances: A Two-Arm Parallel-Group Randomized Controlled Clinical Trial

**DOI:** 10.7759/cureus.73449

**Published:** 2024-11-11

**Authors:** Lana Hasan Albardawel, Kinda Sultan, Mohammad Y. Hajeer, Mohammad Maarouf

**Affiliations:** 1 Department of Orthodontics, Faculty of Dentistry, University of Damascus, Damascus, SYR; 2 Department of Microbiology and Biochemistry, Faculty of Pharmacy, University of Damascus, Damascus, SYR

**Keywords:** fixed orthodontic appliance, gingival index (gi), lactobacillus reuteri, oral health, orthodontic treatment, papillary bleeding index (pbi), periodontal indices, plaque index (pi), probing depth (pd), probiotics

## Abstract

Background and objectives: Fixed orthodontic appliances interfere with daily oral care procedures, causing more plaque accumulation and thus increasing the risk of periodontal diseases. Probiotics have been suggested to maintain oral health using beneficial bacteria. However, the evidence to determine the clinical benefits of probiotics as a supplement to oral health in orthodontic patients is still insufficient. This trial aimed to investigate the protective effect of probiotics on oral health and prevent the development of inflammation of the gingiva and periodontal tissues during orthodontic treatment using fixed orthodontic appliances.

Materials and methods: This was a single-blinded, single-center, two-arm randomized controlled trial conducted between March 2023 and June 2024. A total of 50 patients (13 males (26%) and 37 females (74%) with a mean age of 21.33±2.04) who required a non-extraction-based treatment using fixed orthodontic appliances were enrolled and randomly divided into two groups: the probiotics group (PG) who underwent orthodontic treatment with probiotic tablets of *Lactobacillus reuteri* and the control group (CG) who underwent orthodontic treatment without probiotics. The following periodontal parameters were recorded: plaque index (PI), gingival index (GI), papillary bleeding index (PBI), and probing depth (PD). These indices were assessed three times: before the start of orthodontic treatment (T0) and after three months (T1) and after six months (T2) of applying fixed appliances.

Results: The mean values for PI, GI, PBI, and PD were statistically significantly lower in the PG compared to the CG at both time points T2 and T3 (p<0.05). In the PG, there was a significant difference in the PI between time points T0 and T1 (p<0.001), but there was no significant difference between T1 and T2 (p=0.074). The GI, PBI, and PD differences were also insignificant (p>0.05). Conversely, all studied periodontal parameters significantly increased during the assessment periods in the CG (p<0.001).

Conclusion: Using probiotic lozenges that contain *Lactobacillus reuteri*, alongside regular teeth brushing, during the first six months of fixed orthodontic treatment in adults can help maintain good oral hygiene. This approach reduces plaque buildup and helps prevent the onset of clinical signs associated with periodontal disease.

## Introduction

Orthodontic treatment is essential to manage malocclusion cases and improve the relationship between occlusion and jaw, masticatory function, and facial esthetics [1]. Furthermore, the prevalence and need for orthodontic treatment have increased for therapeutic and aesthetic reasons [2]. 

The lengthy orthodontic treatment with fixed appliances may facilitate dental biofilm accumulation and cause difficulty in brushing [3]. Additionally, orthodontic brackets, bands, ligatures, and wires, which consist of the main fixed appliance components, reduce the physiological mechanism of self-cleaning by the tongue or cheeks, increasing bacterial plaque retention and changing the bacterial population [1]. However, scientific evidence has demonstrated that patients undergoing orthodontic treatment with fixed appliances show qualitative and quantitative changes in the oral microbiome. This change is due to increased supra- and subgingival bacterial plaque retention throughout treatment [4,5]. Additionally, the alteration in the microbial environment following the placement of fixed orthodontic appliances is associated with increased gingival inflammation [5]. An experimental study found a correlation between the levels of salivary pro-inflammatory cytokines, gingival health status, and oral microbial loads in patients receiving orthodontic treatment [6]. Therefore, there has been a high recommendation for better oral hygiene programs to prohibit harmful risks to periodontal and gingival tissues during orthodontic treatment [7]. A new method in dentistry for maintaining oral health and prohibiting periodontal disease using beneficial bacteria has been introduced, known as probiotics [7,8]. Probiotics are living microorganisms that confer a health benefit to the host when ingested in sufficient quantities (in food or as a dietary supplement). The mechanism of action of probiotic bacteria in maintaining oral health competes against oral pathogens for nutrients, growth factors, and adhesion sites [7]. In addition, probiotics can modulate immune inflammatory responses by producing biologically active substances, such as bacteriocins or organic acids [9]. Probiotic strain administration has been suggested to be useful in preventing and treating dental caries and periodontal disease associated with changes in oral microbiome composition and biofilm formation [10-12]. Lately, several papers have investigated the effects of probiotics in enhancing oral health in orthodontic patients, but, up to date, their effectiveness remains controversial [13]. Some studies found that probiotics improve oral health in patients with fixed orthodontic appliances and reduce the pathogenic bacteria counts in saliva and/or dental plaque [14-18].

On the contrary, some studies found insignificant effects of probiotics regarding gingival inflammation [7,19,20] and changes in periodontal pathogen's level [20]. Several systematic reviews (SRs) have recently been conducted to evaluate probiotics' role in orthodontic practice. They showed controversial findings regarding the effect of probiotics on oral health maintenance in orthodontic patients [13,21,22]. Hadj-Hamou et al., from the final qualifying studies, concluded that probiotics in fixed appliance orthodontic patients did not affect the development of gingivitis and enamel decalcification [21]. On the contrary, Pietri et al. found that probiotics facilitate the maintenance of oral health in patients undergoing fixed orthodontic treatment by significantly decreasing the cariogenic bacteria counts in the saliva and oral biofilm [22]. In the same context, Chen et al. concluded that the evidence to determine the probiotics' clinical benefits as an oral health supplement for orthodontic patients is insufficient [13]. Additionally, they reported methodological flaws in previous studies as they lacked standardization in probiotic protocols, leading to variations in strains, concentrations, intervention periods, and follow-up periods [13]. However, the previous three SRs mentioned the need for well-designed randomized controlled trials (RCTs) [13,21,22].

Therefore, the current randomized controlled clinical trial aimed to investigate the protective effect of probiotics on oral health and prevent the development of inflammation of the gingiva and periodontal tissues during orthodontic treatment using metal fixed orthodontic appliances.

## Materials and methods

Study design, registration, and settings

This study was a single-centered, two-arm parallel-group RCT. The trial was conducted between March 2023 and June 2024 in the Department of Orthodontics at the Faculty of Dentistry, University of Damascus, whereas the bacterial analysis and the real-time polymerase chain reaction (PCR) were performed in the Diagnostic Laboratories Department of the Faculty of Pharmacy at the University of Damascus. The trial was registered in the ClinicalTrials.gov database (ID: NCT06641960). Ethical approval was obtained from the Local Research Ethics Committee of the Faculty of Dentistry, University of Damascus (approval number: UDDS-528-19112020/SRC-1810). The University of Damascus funded the trial (Ref no: 501100020595).

Sample size calculation

Sample size calculation was conducted using Minitab® Version 20.4 (Minitab Inc., State College, Pennsylvania, United States). The calculation was performed with a test power of 95%, α=5%, and the assumption that the least significant difference to be detected between the two groups in plaque index (PI) 0.5 (from a previous paper [23]) with a standard deviation of 0.42 (from a previous paper [7]). The previous assumptions were applied with a two-sample t-test; it was found that 20 patients in each group were required. In anticipation of any withdrawal or non-compliance during follow-up, the sample size was increased by 25% to include 50 patients, with 25 patients in each group.

Patients' recruitment and eligibility criteria

The recruitment of patients was carried out at the Department of Orthodontics, Faculty of Dentistry, University of Damascus. Patients who met the following criteria were included: (1) adult patients aged between 18 and 25 years; (2) class I, II, and III malocclusions according to the Angel classification and camouflage treatment which requires alignment of teeth on the upper and lower arches without extraction during the first six months of orthodontic treatment; (3) mild to moderate crowding ≥3 mm on the maxillary and mandibular; (4) good oral health, which was clinically judged by the following: periodontal pocket depth not exceeding 4 mm, no radiographic evidence of bone resorption, PI ≤1, and gingival index (GI) ≤1 [24]; and (5) no previous orthodontic treatment. In contrast, the following exclusion criteria were adopted: (1) the presence of loss of any of the upper or lower permanent teeth (except the third molars); (2) patients who suffer from any systemic disorder that may affect the health of the periodontal tissues or response to treatment (hypertension or diabetes, immune disorders, epilepsy); (3) patients who regularly take medications such as antibiotics, blood pressure medications, analgesics, hormonal medications, tranquilizers, and anticonvulsants during the six months preceding the clinical examination; (4) patients who had allergic to one of the components of the probiotic tablets; (5) patients who undergone treatment for periodontitis during the six months preceding the clinical examination; (6) smokers; (7) pregnant or breastfeeding women; and (8) regular use of antibacterial mouthwash. However, all patients who agreed with the trial criteria were provided adequate information about the nature of the trial, and written informed consent was secured before enrollment in the study.

Randomization, allocation concealment, and blinding

Patients were randomly assigned using the block randomization method. An MSc student in the Department of Orthodontics, not involved in this study, created a randomized patient list using Minitab®. The patients were randomly assigned to five random permuted blocks (i.e., each block contains 10 patients, including two patients from each group) with a 1:1 allocation ratio to one of the following two groups: the first group (the probiotics group (PG)) are those who underwent orthodontic treatment with the use of probiotics, whereas the second group (the control group (CG)) are those who underwent orthodontic treatment without the use of probiotics. Each patient was given a serial number, with the allocation concealed from the researcher to avoid selection bias, by placing the names of the patients and the groups to which they would belong in closed opaque envelopes that were not opened until the stage of application of the fixed orthodontic appliances. This was a single-blind trial, with blinding limited to both stages of measurement (i.e., data collection) and statistical analysis.

Intervention

Two weeks before the application of orthodontic appliances, dental prophylaxis was performed for each patient in both groups. This included scaling, polishing, and instruction on brushing their teeth twice daily. All patients received the same toothpaste and toothbrushes (Colgate-Palmolive®, Mumbai, India) and a leaflet containing information on brushing their teeth regularly using the Bass technique [25].

After two weeks of maintaining proper oral hygiene, patients were instructed to arrive for their appointment in the morning without brushing their teeth for 12 hours [26], ensuring an accurate assessment of periodontal parameters. After that, the leveling and alignment phase was started using MBT 0.022-inch brackets (Pinnacle™, Ortho Technology®, Florida, United States), and the wire sequence was followed up until reaching a 0.019×0.025-inch stainless steel (SS) wire (JISCOP, Gunpo-si, Gyeonggi-do, Korea) which was considered the basal archwire. Subsequently, all patients were instructed to follow oral care guidelines by brushing their teeth twice daily. They were also provided reinforcement oral hygiene messages and videos during follow-up appointments held every three to four weeks. Participants were assigned to the study groups as follows:

The Experimental Group: The PG

Patients in the intervention group were provided with probiotic tablets (Prodentis®, BioGaia AB, Stockholm, Sweden) with recommendations according to the manufacturer [27,28], in addition to following oral hygiene instructions. They were instructed to consume one tablet daily (after dinner and brushing their teeth) for six months. The tablet consumption was started on the day of the fixed orthodontic appliance fitting by placing a tablet on the tongue and letting it dissolve for five minutes without chewing or swallowing. Patients were also instructed not to brush their teeth or rinse their mouths for one hour after taking the tablet and were asked to avoid using antibiotics or antiseptic mouthwashes. Moreover, they were asked to store the tablets below 25°C after opening the package. Patients were given a new package of probiotic supplements during follow-up appointments. A daily reminder was set on each patient's mobile calendar to ensure they took the lozenge on time as instructed. Moreover, between follow-up sessions, patients were sent encouraging pictures and videos to adhere to regularly taking the probiotic dose.

The CG

Patients were asked to maintain good oral hygiene by using only a toothbrush and toothpaste during all orthodontic treatment.

Outcome measures

The periodontal parameters were evaluated at the following time points: (T0) after the oral hygiene phase, before the application of fixed orthodontic appliances; (T1) three months after the application of fixed orthodontic appliances (during the alignment phase); and (T2) six months after the placement of the fixed appliances (at the end of the alignment phase, after the basal archwire was placed). Moreover, at each assessment time, the patients were asked not to brush their teeth for 12 hours (overnight plaque formation) before the indices were recorded [26].

Monitoring the condition of periodontal tissues was based on evaluating the following clinical indices: PI, GI, papillary bleeding index (PBI), and probing depth (PD). In each case, it was examined the reference Ramfjord's teeth [29,30], which were the right first molar (16), left central incisor (21), and left first premolar (24) on the maxillary, as well as the left first molar (36), left central incisor (41), and right first premolar (44) on the mandibular. Additionally, the maxillary left first molar (26) and mandibular right first molar (46) were also adopted to measure the periodontal parameters.

PI

To assess the PI, the tooth surfaces were first air-dried. Then, using a dental explorer and a mouth mirror, the plaque thickness was evaluated according to Löe and Silness on the following surfaces for each of the examined teeth: mid-buccal, mid-lingual, mesiobuccal, and distobuccal surfaces [24]. For each patient, the PI value was determined by calculating the mean values ​​of the studied teeth.

GI

The GI was evaluated using a mouth mirror and Williams' periodontal probe to assess the severity of gingivitis according to Löe and Silness in the following four sites for each of the examined teeth: the mesiobuccal and the distobuccal papilla, as well as the buccal and the lingual gingival margin [31]. For each patient, the GI value was determined by calculating the mean values ​​of the examined teeth.

PBI

The PBI was assessed using Williams' probe, which was inserted into the gingival sulcus at the papilla based on the mesial aspect. After that, the probe was moved coronally to the tip of the papilla. The probe was then repeated on the distal side of the papilla [32]. For each patient, the PBI value was determined by calculating the mean values of the examined teeth.

PD

Williams' periodontal probe measured the PD in millimeters from the gingival margin to the most sulcus's apical portion. Four readings were recorded for each examined tooth: buccal, distal, mesiobuccal, and distobuccal surfaces. For each patient, the PD value was determined by calculating the mean values ​​of the examined teeth. The reading of PD ≤3 mm was considered normal, while any reading >3 mm was considered a gingival pocket [33].

Statistical analysis

The statistical study used IBM SPSS Statistics for Windows, Version 26.0 (Released 2019; IBM Corp., Armonk, New York, United States). At first, descriptive statistics were calculated for each of the study variables. Then, the normality of the data distribution test was carried out using the Shapiro-Wilk test. The chi-squared test was used to detect any significant differences between the two study groups concerning gender. Independent samples t-test was used to compare the patients' ages between the two study groups. The Mann-Whitney U test was applied to compare the amount of change in the periodontal indices between the two study groups at each time separately. After that, the Friedman test was used to compare the three study times within each group separately, and the Wilcoxon signed-rank matched-pairs test was used for post-hoc comparisons. However, a 95% confidence level was adopted in all previous statistical analyses, and thus, a significance level of 0.05 was used to determine the presence of statistically significant differences.

## Results

Patient recruitment, follow-up, entry to data analysis, and baseline sample characteristics

**Figure 1:**
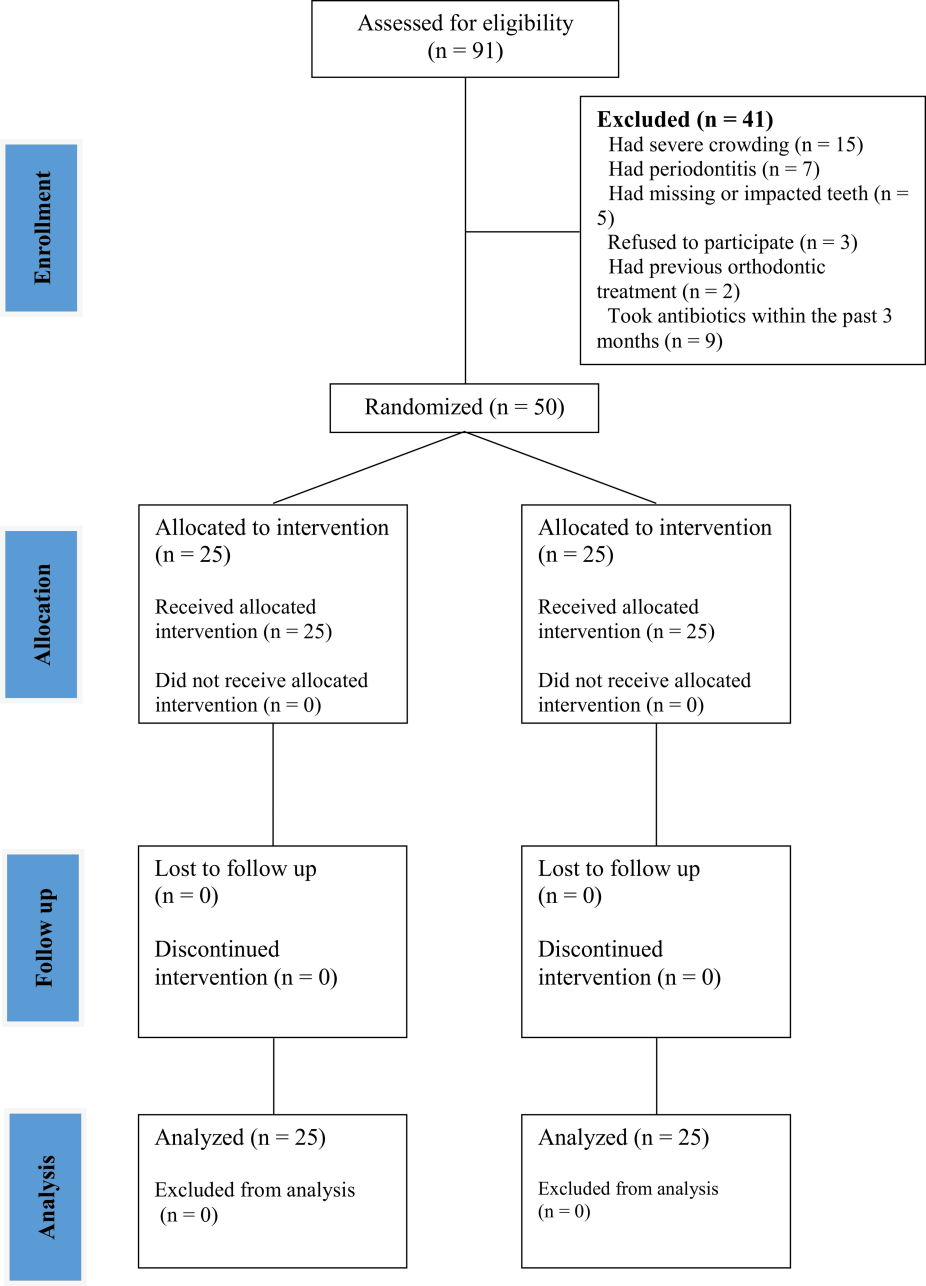
The CONSORT flow diagram of patient recruitment, follow-up, and entry into data analysis. CONSORT: Consolidated Standards of Reporting Trials

Fifty patients (13 (26%) males and 37 (74%) females, with a mean age of 21.33±2.04 years) were included in this research. During the trial period, no dropout occurred; hence, all the patients were included in the statistical analysis. The Consolidated Standards of Reporting Trials (CONSORT) flow diagram is presented in Figure 1. Moreover, the baseline sample characteristics are shown in Table 1. However, when investigating the differences in the average age of the mean age and the gender distribution between the two study groups, no statistically significant differences were found (p=0.325 and p=0.333, respectively; Table 1).

**Table 1:** Baseline sample characteristics regarding gender and age. †: employing chi-squared test; ‡: employing independent samples t-test; N: number of patients; SD: standard deviation

Group	N	Gender	N (%)	P-value†	Mean age (SD)	P-value‡
Probiotics group	25	Male	8 (32%)	0.333	21.62±2.14	0.325
		Female	17 (68%)			
Control group	25	Male	5 (20%)		21.04±1.94	
		Female	20 (80%)			
All sample	50	Male	13 (26%)		21.33±2.04	
		Female	37 (74%)			

Main outcomes

Before applying fixed orthodontic appliances (i.e., T0), no statistically significant differences were found between both groups in all the studied indices (p>0.05; Table 2). Therefore, there was acceptable homogeneity between the two study groups regarding the values of these indices at T0, which indicated that both groups were comparable in all variables.

**Table 2:** Descriptive statistics of the periodontal indices in the two study groups at the three assessment times and the results of the significance tests of comparisons between the two study groups and among the three assessment times in each group. †: employing Mann-Whitney U test to compare means between the two study groups at each assessment time; ‡: employing Friedman test to compare means among the three assessment times in each group separately; n: number of patients; SD: standard deviation; Min: minimum; Max: maximum; PI: plaque index; GI: gingival index; PBI: papillary bleeding index; PD: probing depth; T0: before applying fixed orthodontic appliances; T1: after three months; T2: after six months, *: statistically significant at p<0.05

Variables	Time	Probiotics group (n=25)			Control group (n=25)			P-value†
		Mean (SD)	Min	Max	Mean (SD)	Min	Max	
PI	T0	0.07 (0.17)	0	0.65	0.05 (0.13)	0	0.55	0.923
	T1	0.81 (0.49)	0.25	2	1.14 (0.45)	0	2	0.011*
	T2	1.03 (0.66)	0	2	1.84 (0.20)	1.38	2	<0.001*
	P-value‡	<0.001*			<0.001*			
GI	T0	0 (0)	0	0	0 (0)	0	0	1.000
	T1	0.01 (0.07)	0	0.38	0.63 (0.25)	0.25	1	<0.001*
	T2	0.04 (0.17)	0	0.88	1.48 (0.24)	1	2	<0.001*
	P-value‡	0.368			<0.001*			
PBI	T0	0 (0)	0	0	0 (0)	0	0	1.000
	T1	0 (0)	0	0	0.27 (0.18)	0	0.75	<0.001*
	T2	0 (0)	0	0	0.66 (0.24)	0.25	1	<0.001*
	P-value‡	-			<0.001*			
PD	T0	1.43 (0.30)	1	2.13	1.32 (0.26)	1	2	0.187
	T1	1.48 (0.38)	1	2.63	2.37 (0.76)	1.25	3.38	<0.001*
	T2	1.63 (0.52)	1	3	2.70 (0.72)	3.75	1.63	<0.001*
	P-value‡	0.417			<0.001*			

The descriptive statistic showed that the mean PI value for the PG was 0.07 at baseline (T0) and increased to 1.03 after six months of fixed appliance application (T2). The mean PI value for the CG was 0.05 at the baseline and increased to 1.84 at T2 (Table 2). Regarding GI, no gingivitis was recorded in both groups at T0 (x ®=0; Table 2), whereas the mean GI values were increased at T2 to 0.04 in the PG and to 1.48 in the CG (Table 2). On the other hand, no papillary bleeding was recorded in the three assessment times in the PG and only at T0 in the CG (x ®=0; Table 2). However, after six months of fixed appliance application, the mean PBI value reached 0.66 in the CG (Table 2). The PD remained normal for the three assessment times (1-3 mm) in both groups (Table 2). When comparing the differences between the two study groups after three and six months of fixed appliance application (i.e., T2 and T3), the mean values of PI, GI, PBI, and PD were statistically significantly lower in the PG compared with the control one (p>0.05; Table 2).

Changes within groups

In the PG, Friedman's analysis showed that there was a significant difference between the three assessment time points only regarding PI (p<0.001; Table 2), whereas the differences were insignificant regarding the GI, the PBI, and the PD (p>0.05; Table 3). On the other hand, statistically significant differences were found between the three assessment time points in all the periodontal parameters (p<0.001; Table 2) within the CG.

**Table 3:** Results of the significance testing in pairwise comparisons between the three assessment time points regarding the periodontal indices in each group. †: employing Wilcoxon signed-ranks matched-pair tests; PI: plaque index; GI: gingival index; PBI: papillary bleeding index; PD: probing depth; T0: before applying fixed orthodontic appliances; T1: after three months; T2: after six months; *: statistically significant at p<0.05

Time	Probiotics group	Control group			
	PI	PI	GI	PBI	PD
T0-T1	<0.001*	<0.001*	<0.001*	<0.001*	<0.001*
T1-T2	0.074	<0.001*	<0.001*	<0.001*	<0.001*
T2-T3	<0.001*	<0.001*	<0.001*	<0.001*	<0.001*

Post-hoc pairwise comparisons revealed a statistically significant difference between each of the two different evaluation times regarding the PI in the PG (p<0.001; Table 3), except between times T1 and T2, where there was no significant difference (p=0.074; Table 3). On the other hand, statistically significant differences between the two evaluation times were found in all studied periodontal indices in the CG (p<0.001; Table 3).

Harms

The present study was not associated with any injury recorded in sample members.

## Discussion

This study was designed based on the suggestions of the most recent SRs, which noted problems in the methodology of the studies and differences in the protocol of the previous work steps [13,34,35]. This led to different results for the effect of probiotics on the periodontium. The suggestions of the SRs were to address these methodological problems, which is important to ensure the credibility and appropriateness of the research results. Future studies need to concentrate on discovering the most effective methods of administration, appropriate probiotic strains, and ideal concentrations, as well as increasing the duration of follow-up assessments. Additionally, it is essential to explore the effects of probiotics on oral health by applying microbiological and clinical assessments of individuals receiving orthodontic treatment with different kinds of removable and fixed appliances [13,34,35]. Therefore, this two-arm RCT was conducted to investigate the probiotic effect on oral health and the prevention of the development of inflammation of periodontium during fixed orthodontic treatment.

This study included adult patients (18-25 years old) and excluded young patients due to the influence of growth factors and changes of both metabolic and hormonal on the variables studied in the research (i.e., assessment of periodontal indices), which may lead to incorrect interpretations of the study results [36]. Additionally, older ages were avoided to make the general and oral health status effects similar among the sample patients.

The probiotic *Lactobacillus *strain *L. reuteri* (Prodentis®, BioGaia AB, Stockholm, Sweden) was chosen as several SRs reported in their conclusions the effectiveness of *Lactobacillus* in treating periodontal diseases and reducing the number of bacteria causing them [37-39].

The current study showed that probiotics were effective in maintaining oral health in fixed orthodontic patients during the first six months following the onset of treatment. The use of *L. reuteri* as a probiotic significantly reduced the amount of plaque accumulation in the PG compared to the control one. This can be attributed to the antiplaque properties of the probiotic in several ways, such as reducing the adhesion of bacteria to the tooth surface, inhibiting the growth and reproduction of bacteria on the tooth surface, and modifying the biochemistry of this plaque to reduce the formation of toxic products for bacteria [40].

Gingivitis scores in the PG showed a significant decrease compared to the CG. Our finding was in accordance with the study of Shah et al. [15]. In contrast, Benic et al. [19] and Habib [20] found no significant differences between the PG and CG regarding the GI. This may be attributed to the difference in the type of probiotics applied, as the probiotic of the *Lactobacillus* type was used in the current study, while in the previous two studies, another type was used (i.e., *S. salivarius*) [19,20]. Another reason for this difference may be the variance in the dose of the probiotic and the application period. In the study of Habib [20], two tablets were given twice daily for the first seven days and then two tablets once daily for 21 days. On the other hand, in the study of Benic et al. [19], one tablet was given twice daily for a month. The present study instructed patients to consume one tablet daily for six months.

After three and six months of orthodontic treatment, the administration of *L. reuteri *in the PG was accompanied by no papillary bleeding, while minor bleeding was recorded in the CG, with a significant difference between the two groups. Previous studies reported similar findings [7,28].

The current study showed increased PD after three and six months of orthodontic treatment. Although the average PD in both groups remained within the limits of the normal sulcus depth, the increase in the sulcus depth in the CG was significantly greater. After reviewing the published literature, we did not find any study that evaluated the effect of probiotics on PD in fixed orthodontic patients to compare our findings.

According to the present results, plaque accumulation increased significantly in the PG during the first three months of fixed appliance application. This may be because patients during the first period after orthodontic appliance application found it difficult to perform oral care procedures well [5]. On the other hand, the periodontal indices' scores in the CG increased significantly after three and six months of fixed orthodontic appliance application. This increase is likely because fixed appliances promote food particle retention and provide plaque retention sites, making preservation of oral hygiene more difficult and increasing the risk of gingivitis and periodontitis [5], increasing the corresponding periodontal index values. Our results were consistent with previous studies, which found that treatment with fixed orthodontic appliances was associated with an increase in the scores of PI [30,41], GI, PBI, and PD [30,42].

Limitations of the current work

In the present study, the evaluation of the effect of probiotics was limited to clinical periodontal indices, and their effect on oral bacterial counts was not evaluated. In addition, the evaluation period was limited to the first six months of fixed orthodontic appliance application. Furthermore, future research should consider the effect of probiotics on oral health in patients who will undergo long-term orthodontic treatment, such as extraction-based treatment.

## Conclusions

Taking into account the limitations of this study, we can conclude that the use of *L. reuteri* probiotic lozenges in conjunction with regular tooth brushing during the initial six months of orthodontic treatment for adults undergoing fixed metal appliance therapy was beneficial in maintaining oral hygiene. This proactive measure effectively minimized the significant increase in plaque accumulation and the clinical indicators of periodontal disease. In contrast, the CG, which practiced only regular tooth brushing, showed a notable and significant increase in all measured indices, including the PI, GI, PBI, and PD, at designated evaluation points throughout the treatment period. This difference highlights the importance of incorporating probiotic lozenges in the oral care routine during orthodontic treatment to promote better oral health outcomes.
